# Structural differences of cell walls in earlywood and latewood of *Pinus sylvestris* and their contribution to biomass recalcitrance

**DOI:** 10.3389/fpls.2023.1283093

**Published:** 2023-12-08

**Authors:** Aleksandra Liszka, Raymond Wightman, Dariusz Latowski, Matthieu Bourdon, Kristian B. R. M. Krogh, Marcin Pietrzykowski, Jan J. Lyczakowski

**Affiliations:** ^1^ Department of Plant Biotechnology, Faculty of Biochemistry, Biophysics and Biotechnology, Jagiellonian University, Krakow, Poland; ^2^ Jagiellonian University, Doctoral School of Exact and Natural Sciences, Krakow, Poland; ^3^ Microscopy Core Facility, Sainsbury Laboratory, University of Cambridge, Cambridge, United Kingdom; ^4^ Department of Plant Physiology and Biochemistry, Faculty of Biochemistry, Biophysics and Biotechnology, Jagiellonian University, Krakow, Poland; ^5^ Sainsbury Laboratory, University of Cambridge, Cambridge, United Kingdom; ^6^ Department of Protein Biochemistry and Stability, Novozymes A/S, Bagsværd, Denmark; ^7^ Department of Ecological Engineering and Forest Hydrology, Faculty of Forestry, University of Agriculture in Krakow, Krakow, Poland

**Keywords:** Pinus sylvestris, cell wall, earlywood, latewood, hemicelluloses, lignin, recalcitrance

## Abstract

Scots pine (*Pinus sylvestris* L.) is an evergreen coniferous tree with wide distribution and good growth performance in a range of habitats. Therefore, wood from *P. sylvestris* is produced in many managed forests and is frequently used in industry. Despite the importance of pine wood, we still do not fully understand its molecular structure what limits improvements in its processing. One of the basic features leading to variation in wood properties is the presence of earlywood and latewood which form annual growth rings. Here, we characterise biochemical traits that differentiate cell walls of earlywood and latewood in Scots pine. We discover that latewood is less recalcitrant to enzymatic digestion, with galactoglucomannan showing particularly pronounced difference in accessibility. Interestingly, characterisation of lignin reveals a higher proportion of coniferaldehydes in pine latewood and suggests the presence of a different linkage landscape in this wood type. With complementary analysis of wood polysaccharides this enabled us to propose the first detailed molecular model of earlywood and latewood and to conclude that the variation in lignin structure is likely the main determinant of differences in recalcitrance observed between the two wood types in pine. Our discoveries lay the foundation for improvements in industrial processes that use pine wood since we show clear pathways for increasing the efficiency of enzymatic processing of this renewable material. Our work will help guide future breeding of pine trees with desired timber properties and can help link molecular structure of softwood cell walls to function of the different types of xylem in conifers.

## 1 Introduction

Scots pine (*Pinus sylvestris*) is a coniferous species with major importance for a range of ecosystems and industries. It has the greatest distribution of all pine species (Eckenwalder, 2009) with growth, in Europe where it is native, in locations ranging from northern Scandinavia down to the Mediterranean. Because of its good growth performance in a range of ecosystems (Skilling, 1990) and on marginal lands the species is frequently used in managed forests, for example, in reclamation and reforestation of post-industrial areas (Pietrzykowski and Socha, 2011; Vacek et al., 2021). For these reasons, Scots pine is one of the major forestry species and therefore, pine wood finds application in numerous industrial sectors (Parciak, 2013; Houston Durrant et al., 2016) such as construction, paper and pulp production and furniture manufacturing. Moreover, there is growing interest to use pine wood as a feedstock for novel technological processes such as biofuel fermentation or manufacturing nanoporous films (Ray et al., 2010; Funda et al., 2020; Bettotti and Scarpa, 2022). Unfortunately, improvements in the application of pine timber are hindered by the poor understanding of its molecular structure. To address this issue, we evaluated here the structural differences in cell walls of earlywood and latewood of pine. Presence of these two wood types in softwood, wood from coniferous trees, is one of the main sources of variation in its properties and performance (Perré and Turner, 2001) but there is little information available on the biochemical and molecular reasons for the observed differences.

Most coniferous plants, including *P. sylvestris*, growing in temperate and boreal climates have a specific annual growth pattern. Xylem deposited in spring, at the start of the growth season, is known as earlywood (EW) and characterised by the presence of tracheids with a thin cell wall and a wide lumen. In summer, coniferous species transition to deposit tracheids with greatly thickened cell walls at the expense of a smaller lumen (Ramage et al., 2017), referred to as latewood (LW). The seasonal progression from one type of wood to the other leads to the formation of so called “growth rings” frequently used in environmental studies and is liked to increased need for water transport in spring which is facilitated through EW. Regardless of the morphological differences between EW- and LW-forming cells, both types of timber are almost entirely formed from plant secondary cell walls – an extracellular matrix made primarily from cellulose, hemicellulose and lignin (Schweingruber, 2007). Importantly, differences between molecular architecture of cell walls of EW and LW likely impact the properties of the two types of woody material and is therefore of key importance for the physiological function of the two wood types and also for their industrial utilisation.

Cellulose is the main polysaccharide of softwood cell walls, accounting for about a third of the material (Sjöström and Alen, 1999). At the molecular level, cellulose has a simple repeating structure of β-1,4-linked glucopyranosyl residues, forming glucan chains that coalesce into a cellulose microfibril(Fernandes et al., 2011). The exact structure of the microfibril is unknown, however, it has been suggested that the elementary microfibril consists in the order of 18 individual glucan chains (Gonneau et al., 2014; Hill et al., 2014; Kubicki et al., 2018). Galactoglucomannan (GGM) and xylan are the principal hemicelluloses in softwood, with the first one contributing about 20% of cell wall material and the latter one about 10% (Scheller and Ulvskov, 2010). GGM has a backbone formed from both β-1,4-linked mannosyl and glucosyl residues with some mannosyl residues substituted by an α-1,6-linked galactosyl branch. The softwood glucomannan backbone is also often acetylated (Gille and Pauly, 2012). Xylan is a polymer of β-1,4-linked xylopyranosyl residues (Scheller and Ulvskov, 2010). All xylans carry α-1–2 linked glucuronic acid (GlcA) branches which in softwood are largely methylated on carbon 4, leading to formation of 4-O-Methyl-glucuronic acid (MeGlcA). In addition to MeGlcA decorations, softwood xylan carries α-1,3–linked arabinofuranosyl decorations (Busse-Wicher et al., 2016a). Lignin, accounting for about a third of softwood cell walls, is a polyphenolic hydrophobic compound which impregnates wood. Softwood lignin, formed through coupling of monolignol radicals in tracheid secondary cell walls, is composed mainly of coniferyl alcohol molecules bonded primarily through different types of ether linkages (Vanholme et al., 2010). Taken together, the constituents that form the secondary wall matrix provide mechanical strength for an upwards growth habit and to resist the large negative pressures associated with water transport in the xylem. However, their assembly and interaction, which dictate cell wall properties, is not fully understood and likely varies between different types of wood, such as EW and LW of softwood.

In the cell wall, polysaccharides and lignin assemble into structures known as macrofibrils (Donaldson, 2007; Lyczakowski et al., 2019), which are 30 nm in diameter on average in softwood. Within these macrofibrils cell wall polysaccharides and lignin interact through covalent and non-covalent bonding (Kang et al., 2019; Terrett and Dupree, 2019; Gao et al., 2020; Kirui et al., 2022). For example, recent solid state NMR analysis indicated that softwood xylan and GGM associate with the cellulose microfibril, likely at least partially through hydrogen bonding (Terrett et al., 2019). Lignin localises close to this multi-polysaccharide moiety and its association may be mediated, for example, through ether bonding with the GGM (Nishimura et al., 2018) or the proposed ester link with the MeGlcA of xylan (Oinonen et al., 2015; Terrett and Dupree, 2019). Importantly, the precise structure of the cell wall polysaccharides influences their capacity for intermolecular interactions (Grantham et al., 2017; Martínez-Abad et al., 2017; Yu et al., 2018; Martínez-Abad et al., 2020), which then impact plant biomass assembly (Crowe et al., 2021) and properties. This means the chemical structure of EW and LW cell wall components is likely to have a significant effect on industrially relevant features of softwood. Despite that, the diversity in the structure of cell wall elements between EW and LW remains largely unknown what is a key obstacle in improvement of processes using timber. The importance of this topic is further accentuated by the fact that currently many managed forest plantations utilise short rotation cycles which produce large quantities of juvenile wood, which, unlike mature wood, is composed mainly of EW (Clark et al., 2006; Cown and Dowling, 2015). Disproportion between the EW and LW content in juvenile wood may be one of the reasons for its poor performance in industrial applications (Cown and Dowling, 2015). This further highlights the need to understand the molecular structure of the two wood types.

To address these issues, here we analyse the composition, structure and properties of polysaccharides and lignin which form secondary cell walls of *P. sylvestris* EW and LW. Our data indicate that, the LW cell walls are significantly more accessible to enzymatic digestion than the EW ones. Detailed characterisation of the hemicellulose and lignin structure allowed us to conclude that this difference likely originates from the variation in the lignin structure observed between EW and LW of pine. We integrate here our results with previously published data to propose a detailed model showing the molecular architecture of pine’s earlywood and latewood cell walls which may account for differences in accessibility to enzymatic digestion. Our model may guide development of enzymatic approaches to wood deconstruction, in particular EW-rich juvenile wood deconstruction, which are a key step in the sustainable use of this material. Since our analysis is based on cores isolated from many *P. sylvestris* individuals, our data also highlights the diversity in the cell wall structure in EW and LW of this species. We think this diversity may form the basis for breeding projects aimed at isolation of pine genotypes depositing wood with desired properties.

## 2 Materials and methods

### 2.1 Plant material and sampling site

In order to characterise the plant cell wall structure in softwood earlywood (EW) and latewood (LW), we isolated cores from *P. sylvestris* trees. Cores were air dried and rings corresponding to EW and LW were sectioned under a stereomicroscope for each analysed tree (**Figure S1**). Between 20 and 30 EW or LW sections were pooled together to obtain enough cell wall material for each studied individual.

Cores were isolated from thirty pine individuals growing in Niepołomice Forest (Polish: Puszcza Niepołomicka), which is a large forest complex in western part of Sandomierz Basin, about 20 km east of Kraków (Southern Poland). Exact geographical coordinates of the collection area are 50°00’51.785’’ N, 20°37’05.737’’ E. All of sample pine trees growing on similar habitats conditions on fresh coniferous forest sites developed on sandy soils. The cores were obtained from pines between 80 and 90 years of age. Earlywood and latewood sections were isolated from individual cores through sectioning with a scalpel under a stereomicroscope (Opta-Tech see **Figures S1A-C**). Alcohol insoluble residues were prepared for each EW and LW sample using a previously describe method (Mortimer et al., 2010).

### 2.2 Saccharification of plant biomass

All the saccharification experiments were performed on Alcohol Insoluble Residues (AIR) with minor changes to the previously describe method (Lyczakowski et al., 2017). For better extractability, a 1 mg/mL suspension of AIR in water was heat-treated at 60°C for 6h. After heating, the biomass was desiccated *in vacuo* and the pellet was resuspended in 1 ml of 100 mM ammonium acetate pH 5.5. Saccharification was performed in technical triplicate for each sample type using Cellic CTec2® enzyme. Each reaction was amended with 20 µl of 1:10 Cellic CTec2® dilution in 100 mM ammonium acetate pH 5.0 buffer and the reaction was performed for 24 h at 45°C with 1200 rpm mixing applied for 30 s after every 2.5 minutes. The monosaccharides content was measured using Megazyme D-Xylose (K-XYLOSE) and D-Glucose (K-GLUHK) kits.

Accessibility of mannan was measured by GH5 digestion of AIR. For this experiment, 900 µg of AIR was suspended in 500 µL of 0.1 M ammonium acetate pH 5.5 buffer and digested for 24 hours at 30°C. The reaction was precipitated in 65% ethanol at -20°C overnight to remove any undigested polysaccharide. Following precipitation, the supernatant was removed, desiccated and hydrolased with 2 M trifluoroacetic acid (TFA) for 30 minutes at 120°C. The mannose content in the hydrolysate was quantified with HPLC as described in section 2.3 of Materials and Methods.

### 2.3 Monosaccharide composition analysis and quantification of cellulose content

For monosaccharide analysis and cellulose content quantification 1 mg of AIR was treated with a 500 µl 2 M TFA for 2 h at 120°C. The supernatant was used for monosaccharide analysis of the hemicellulose fraction, which was performed through separation of 3-methyl-1-phenyl-2-pyrazoline-5-one (PMP) derivatised simple sugars on Phenomenex Fusion-RP column mounted on Agilent Technologies 1260 Infinity II HPLC System (method adapted after (Yu et al., 2014)). For the separation, 0.1 M phosphate buffer pH 6.6 mixed with acetonitrile at 82:18 (v:v) ratio was used and flown at 1 mL/min rate. No gradient was used. Annotation of monosaccharides was performed by separation of individual monosaccharides: D-mannose, D-ribose, L-rhamnose, D-galacturonic acid, D-glucose, D-galactose, D-xylose, L-arabinose and L-fucose and of their mixture with 36 nmol of each standard injected (**Figure S1D**). Quantitative analysis of the mannose content was realized by comparing the area of the mannose signal to that obtained for mannose calibration curve generated by injection of different amounts of monosaccharide (3.6 nmol, 7.2 nmol, 18 nmol, 36 nmol, 72 nmol) onto a column.

The pellet left after TFA hydrolysis of AIR was washed with distilled water and used for cellulose quantification. To hydrolyse the crystalline cellulose, dried samples were treated with 5M H_2_SO_4_ for 1 h at room temperature, followed by dilution of H_2_SO_4_ to 1 M concentration and further incubation at 120°C for 3 h. The estimation of cellulose content was performed with anthrone method, according to (Kumar and Turner, 2015) with each sample measured in three technical replicates.

### 2.4 Polysaccharide analysis by carbohydrate gel electrophoresis

PACE was performed with small alterations to previously published protocols (Mortimer, 2017; Lyczakowski et al., 2021). Namely, in this work the analysis was performed with 1 mm thick 10% acrylamide gels on a BioRad Protean IX electrophoresis system under constant 500V voltage. The gels were imaged after 1h of electrophoresis using a BioRad Chemidoc MP imager to visualise 8-AminoNaphthalene-1,3,6-TriSulfonic acid (ANTS) labelled oligosaccharides. All other procedures, buffers and quantification techniques used were the same as previously described (Mortimer, 2017). For the digestion of xylan 15U of *Neocallimastix patriciarum* xylanase GH11 (Megazyme, E-XYLNP) was incubated with 100 µg of NaOH extracted AIR for 24 hours at 37°C. Supplementary digestions of xylan were performed with 1.5 µg of glucuronidase GH115 from *Talaromyces leycettanus* and/or 1.5 µg of arabinofuranosidase GH51 from *Meripilus giganteus* added to the GH11 digestion. GGM was digested from 100 µg of NaOH extracted AIR or 900 µg of unextracted AIR with 2 µg of mannanase GH26 from *Cellvibrio japonicus* or 2 µg of mannanase GH5 from *Talaromyces leycettanus*. GGM digestions were performed for 24 hours at 30°C. Mannanases, glucuronidase and arabinofuranosidase were supplied by Novozymes AS, Denmark.

### 2.5 Confocal microscopy and image analysis

Lignin autofluorescence was recorded using Axio Observer.Z1 inverted microscope (Carl Zeiss, Jena, Germany) equipped with a LSM 880 confocal module. A Plan-Apochromat 10×/0.45NA objective was used to visualize large tissue fragments. Wood cores were cut to obtain cross-sections which reveal a xylem lumen. Sections were treated with 4 M NaOH for 10 seconds and rinsed with water. Lignin autofluorescence was excited with a 405 nm laser and the fluorescence emission was recorded within the range of 410 - 710 nm as the green channel (Decou et al., 2017). Images were acquired as z-stacks (4.5 µm step size) with different numbers of slices depending on the xylem cross-section region assessed. The ratio of lignin content in latewood/earlywood was measured with ImageJ using a dedicated quantification script (**Supporting Information Script 1**).

### 2.6 Raman spectroscopy of tree cores

Raman microscopy was carried out on a Renishaw InVia system using a 785 nm laser forming a line (not spot), 1200 l/mm grating, 5x objective lens and the WiRE acquisition software. Cylindrical cores were immobilised on a slide on the Raman microscope stage and a WiRE surface was recorded thus keeping the core in focus along its length. Illumination was through the lens (epi) where early and latewood zones could be identified. An in-focus Y-line scan was taken along the core (aligned to the X axis) with a step size of 250 microns in the X axis between sample points. Typically, 150-250 points were taken along the core. Acquisition settings were 1400 cm^-1^ centre (corresponding to spectral window of 842 - 1903 cm^-1^), high confocality, 2s exposure, 100% laser power, 2x accumulations. All spectra were baseline subtracted in WiRE software.

High magnification Raman line maps of cell walls at submicron intervals was carried out on a Renishaw Qontor system using a Cobolt 785 nm laser forming a spot through a Leica 100x 0.95NA objective lens. Laser power was set to 50% and a 10s prebleach step was added before each acquisition. Acquisition settings were 2 s exposure and 10x accumulations. Line maps were drawn with a step size set to 0.3 micron.

## 3 Results

### 3.1 Cell walls of pine earlywood and latewood have similar composition but different resistance to enzymatic saccharification

**Figure 1:**
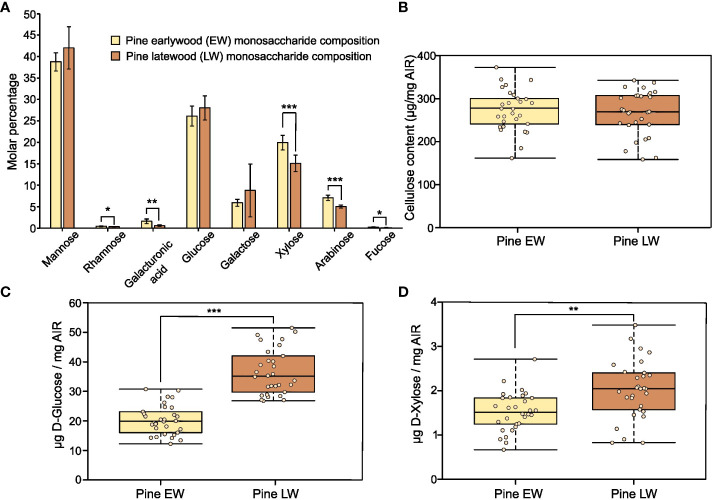
Recalcitrance and composition of pine EW and LW. Throughout the figure light brown indicates EW and dark brown denotes LW. **(A)** Monosaccharide composition analysis of pine EW and LW after TFA hydrolysis of AIR. **(B)** Cellulose content in pine EW and LW. **(C)** D-Glucose and **(D)** D-Xylose release from pine EW and LW after saccharification of heat preatreated AIR with Cellic® CTec2. Each analysed tree is shown as an individual data point on boxplots. For monosaccharide composition analysis, AIR from six trees was analysed. Median is marked in the middle of boxplots in **(B–D)** with rectangle extending to the lower and upper quartile and whiskers showing the minimal and maximal values. Error bars represent standard deviation in **(A)**. Results of two sample Student’s t-test are reported with * marking p ≤ 0.05, ** marking p ≤ 0.01 and *** marking p ≤ 0.001.

To characterise the polysaccharide composition of the EW and LW cell walls we sectioned respective wood parts from tree cores (**Figure S1**) and analysed monosaccharide composition of non-cellulosic polysaccharides of AIR isolated from both sample types (**Figure 1A**). For both EW and LW, mannose and glucose were the principal sugars detected. They account for 70% of all non-cellulosic monosaccharides present in the hydrolysate, suggesting that glucomannan is the dominant hemicellulose in both EW and LW of *Pinus sylvestris*. Galactose was also detected, suggesting that some of the glucomannan present in the material may be branched with galactose substitutions giving rise to galactoglucomannan (GGM). Other sugars present in the TFA-AIR were xylose and arabinose that likely originate from xylan polymers. Interestingly, xylose and arabinose were more abundant in EW than in LW, indicating that xylan likely accounts for a greater proportion of cell wall in the former. Galacturonic acid, rhamnose and fucose were all detectable but their total content did not exceed 3%. This suggests that for both EW and LW, the secondary cell wall polysaccharides account for the majority of the sample and that primary cell wall polysaccharides, such as homogalacturonan and rhamnogalacturonan, represent only a marginal proportion of the studied material. To provide further insight into the composition of EW and LW, we quantified the cellulose content in both sample types (**Figure 1B**). The quantity of cellulose varied across the individuals studied but overall, we did not detect significant difference in the cellulose content between EW and LW of *P. sylvestris.*

To analyse the recalcitrance of the EW and LW cell wall materials we performed saccharification experiments on AIR from both types of wood. In our experiments, we analysed the release of glucose (**Figure 1C**) and xylose (**Figure 1D**) after treatment of biomass with a commercial enzyme cocktail, Cellic® CTec2. For both monosaccharides studied the amount of sugars released was significantly greater from LW than from EW. Given that the glucan content in EW and LW is not significantly different and that EW appears to contain a higher proportion of xylan than the LW, these results show that LW cell walls are more accessible to enzymatic digestion than the EW ones.

### 3.2 Mannan is readily digested in latewood but not in earlywood

**Figure 2:**
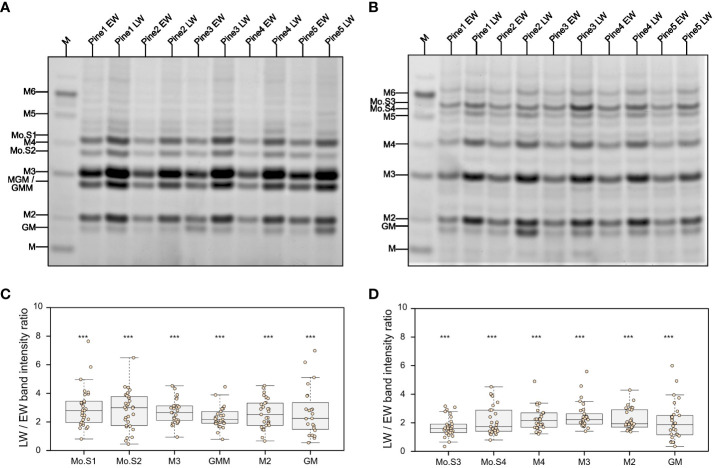
Analysis of mannan structure and accessibility in pine EW and LW through digestion with mannanases and PACE. Results of **(A)** GH5 and **(B)** GH26 mannanase digestion of EW and LW originating from five different pine individuals. M denotes a lane in which a M1 to M6 oligosaccharide standard was loaded. Known mannooligosaccharides are labelled and unknown mannooligosacharides are marked from Mo.S1 to Mo.S4. Intensity ratio between LW and EW oligosaccharides released after mannanase **(C)** GH5 and **(D)** GH26 digestion shown on boxplots. Datapoints for each analysed tree are shown as individual dots on boxplots. Median is marked in the middle of boxplots in **(C, D)** with rectangle extending to the lower and upper quartile and whiskers showing the minimal and maximal values (excluding outliers not included in median quantitation). Results of one sample Student’s t-test are reported with *** marking p ≤ 0.001. Hypothesis tested with one sample Student’s t test assumed intensity ratio equal to 1.

Since our primary saccharification analysis (**Figures 1C, D**) was limited to measurement of glucose and xylose release from EW and LW, we wanted to also compare the recalcitrance of the main softwood hemicellulose, the GGM, to enzymatic digestion in the two wood types. To this end, we performed digestions of AIR with mannanases GH5 (**Figure 2A**) and GH26 (**Figure 2B**). The released oligosaccharides were derivatised with ANTS fluorophore and analysed with PACE. Based on previous publications and comparison to the mannan migration standard, we were able to annotate several structures released by the two enzymes. Mannotriose (M3) followed by MGM or GMM trisaccharide were the dominant products obtained after GH5 digestion. Mannotetraose was also detectable, but its abundance was lower compared to the shorter oligosaccharides. Digestion of GGM from EW and LW with mannanase GH26 resulted in release of longer products than these obtained with GH5 digestions, with some oligosaccharides migrating between the mannohexaose and mannopentaose standards. Interestingly, our analysis did not reveal any major structural differences between the EW and LW mannan, that is the PACE banding pattern obtained after digestion with both mannanase GH5 or GH26 was similar for the two wood types. This suggests that the monosaccharide composition and the distribution of glucose and mannose units in the backbone are similar for EW and LW GGM. Further inspection of PACE gels obtained after GH5 and GH26 digestion indicated that the intensity of all oligosaccharides released by the two enzymes was significantly greater for digests performed using the LW biomass than for EW biomass (**Figures 2C, D**). This difference, consistent across wood samples from all studied tree individuals, may be due to variable GGM content in the two wood types or could be a result of different accessibility of EW and LW GGM within the cell wall matrix.

To evaluate why mannanase digestions released different amounts of oligosaccharides, we performed a quantitative analysis of mannose content in the two wood types. Our mannose content quantification indicates that the amount of the sugar in EW and LW does not differ significantly. On average, mannose accounts for 73 ( ± 13) µg/mg and 81 ( ± 21) µg/mg of AIR in EW and LW respectively. This suggests that the increased accessibility of LW GGM to enzymatic digestion, when compared to EW GGM, may be the main reason for the difference in the amount of mannooligosaccharides observed on PACE. To further evaluate the accessibility of GGM to enzymatic digestion, we performed hydrolysis of EW and LW with mannanase GH5 using AIR not treated with NaOH as a feedstock (**Figure S2**). In line with previous results, LW digestions resulted in the release of a larger quantity of mannooligosaccharides than the EW ones (**Figure S2A**). Moreover, quantitative analysis of the total released mannose with HPLC showed that GH5 hydrolysis results in greater mannan digestion on LW feedstock than on EW one (**Figure S2B**). Taken together, these data shows that LW mannan is more accessible to enzymatic digestion compared to EW. Given that GGM also contains glucose, the higher digestibility of LW GGM may be one reason for higher glucose yield obtained in the saccharification experiments (**Figure 1C**). However, since GGM does not contain xylose the difference in the yield of this monosaccharide in the saccharification experiments (**Figure 1D**) is likely associated with other aspects of EW and LW cell wall molecular architecture.

### 3.3 Small variations in xylan structure do not account for the large differences in recalcitrance

**Figure 3:**
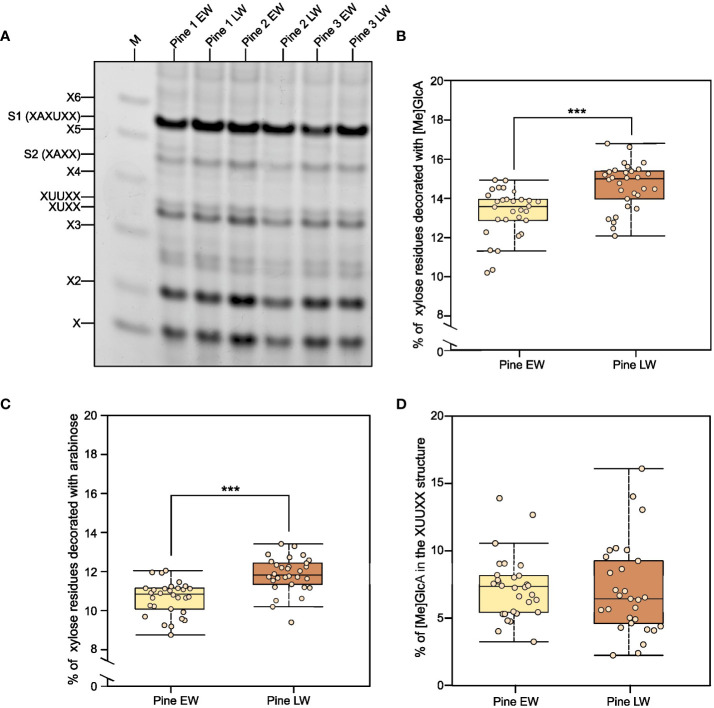
Analysis of xylan structure and branching in pine EW and LW with PACE. **(A)** Results of GH11 xylanase digestion of EW and LW originating from three different pine individuals. M denotes a lane in which a X1 to X6 oligosaccharide standard was loaded. Known glucuronidated and/or arabinosylated oligosaccharides are also labelled. Digestion products are in line with previously described structure of *Pinus nigra* xylan (Busse-Wicher et al., 2016a) and that of *Picea abies* xylan (Martínez-Abad et al., 2017). **(B)** Quantification of the degree of xylan glucuronidation in EW and LW of pine. **(C)** Quantification of the degree of xylan arabinosylation in EW and LW of pine. **(D)** Percentage of xylan glucuronidation contributed by the XUUXX structure specific for conifer xylan. Throughout the figure light brown indicates EW and dark brown denotes LW. Datapoints for each analysed tree are shown as individual dots on boxplots. Median is marked in the middle of boxplots in **(B–D)** with rectangle extending to the lower and upper quartile and whiskers showing the minimal and maximal values (excluding outliers not included in median quantitation). Results of Mann Whitney test are reported with *** marking p ≤ 0.001.

To investigate why EW and LW cell wall polysaccharides show accessibility differences during enzymatic digestion, we started by examining the xylan structure. Evidence from other types of plant biomass indicate that xylan branching with glucuronic acid (GlcA) is critical for the maintenance of biomass resistance to enzymatic degradation (Lyczakowski et al., 2017). In addition to being glucuronidated, conifer xylan also has arabinose branches (Busse-Wicher et al., 2016a). These substitutions may contribute to the maintenance of stable xylan-cellulose interaction (Martínez-Abad et al., 2017; Pereira et al., 2017), and consequently influence the distinct properties of EW and LW secondary cell walls in pine. To assess this, we performed alkali extraction of AIR from both sample types, releasing polysaccharides that were then digested with xylanase GH11. Released oligosaccharides were labelled with the ANTS fluorophore and analysed with PACE (**Figure 3A**). Interestingly, the overall banding pattern for EW and LW were similar, i.e. the same oligosaccharides were released from both sample types. Xylanase GH11 is able to digest unbranched regions of the xylan backbone and is inhibited by the presence of xylan branches (Vardakou et al., 2008; Paës et al., 2012). Compared to the migration standard and previous publications, we were able to annotate several structures released by GH11 from EW and LW xylan. In our digests we detected xylose, xylobiose and xylotriose. We also observed release of both XUXX and XUUXX glucuronidated oligosaccharides (Mortimer et al., 2010; Lyczakowski et al., 2021) (for information on oligosaccharide structures released please see **Figure S3**). In addition, two other main products were observed, which migrated slower than the glucuronidated structures (**Figure 3A**, annotated S1 and S2). Since softwood contains arabinoglucuronoxylan, oligosaccharides S1 and S2 likely contain arabinose decorations on the xylan backbone. As GH11 needs two unsubstituted xyloses on the −1 and −2 sites and one unsubstituted xylose on the +1 site to digest xylan (Vardakou et al., 2008; Paës et al., 2012) (**Figure S3**), oligosaccharides produced by the enzyme contain one unsubstituted xylosyl residue at the non-reducing end, and two unsubstituted xylosyl residues at the reducing end. As such, the other structure likely display two decorations interspaced by just one xylosyl residue. Our additional digests with arabinofuranosidase GH51 and glucuronidase GH115 (**Figure S3A**) enabled us to confirm that, in agreement with previously published information for spruce (Martínez-Abad et al., 2017) and data for monocot arabinoxylan (Tryfona et al., 2019), we can annotate pine structure S1 as being XAXUXX and oligosaccharide S2 as XAXX. (**Figure S3B**).

Having established the identity of the xylan-derived oligosaccharides, we were able to quantify the degree of xylan glucuronidation (**Figure 3B**) and arabinosylation (**Figure 3C**) in the two wood types. Our analysis indicates that the extent to which xylan is arabinosylated and glucuronidated is greater in LW than in EW. The difference, although only just above 1% of the total xylan content, is statistically significant for both types of xylan branching. We also quantified the proportion of xylan glucuronidation that is contributed by GlcA placed on two consecutive xyloses (**Figure 3D** - XUUXX structure described by (Martínez-Abad et al., 2017; Lyczakowski et al., 2021)). This value was the same for EW and LW and, on average, it did not exceed 10% of the total GlcA content. In summary, any differences in xylan structure between EW and LW are minimal and are unlikely to explain the observed difference in the recalcitrance of the two wood types to enzymatic digestion.

### 3.4 Lignin structure differs between EW and LW

Since variation in hemicellulose structure is unlikely to explain the observed difference in the resistance of EW and LW to enzymatic digestion, we wanted to evaluate other aspects of the cell wall molecular architecture in the two wood types. Recalcitrance of woody plant biomass is influenced by the extent of lignification and the exact lignin structure (Van Acker et al., 2013; Yoo et al., 2018). We therefore examined whether lignin may contribute to the observed difference in EW and LW digestibility. Given that our sampling technique provided just a limited amount of EW and LW material from each tree we employed non-destructive techniques for lignin analysis, namely confocal microscopy (Decou et al., 2017) and Raman spectroscopy (Gierlinger et al., 2012; Gorzsás, 2017).

**Figure 4:**
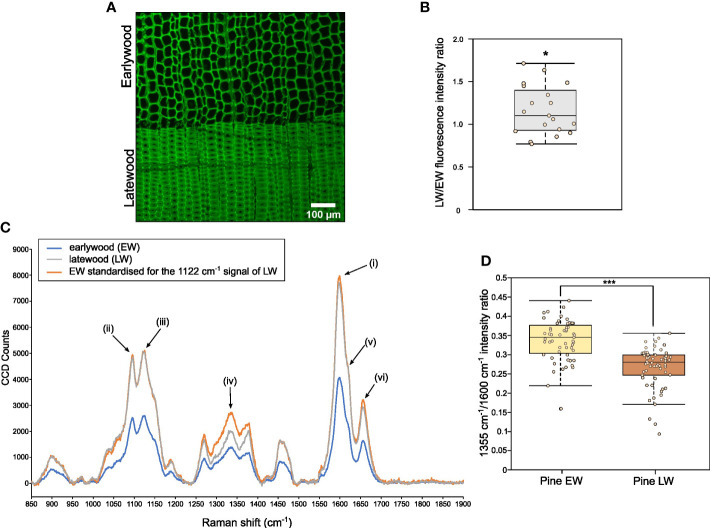
Analysis of relative lignin autofluorescence and structural features in pine EW and LW through confocal microscopy and Raman spectroscopy. **(A)** Confocal image showing lignin autofluorescence in EW and LW of pine. Scale bar is provided. **(B)** Quantification of lignin autofluorescence ratio between LW and EW of pine obtained after analysis of 20 images showing both growth zones. Quantification was performed with a dedicated ImageJ macro. **(C)** Averaged Raman spectrum for pine EW (blue), LW (grey) and for EW with signal intensity standardised to match 1122 cm^-1^ cellulose signals for both wood types (orange). Specific peaks of the Raman spectrum are marked as follows: (i) 1600 cm^-1^ main orientation insensitive lignin signal; (ii) 1095 cm^-1^ cellulose signal; (iii) 1122 cm^-1^ orientation insensitive cellulose signal; (iv) 1335 cm^-1^ signal associated with aliphatic hydroxyls of lignin; (v) 1620 cm^-1^ peak area associated with coniferaldehydes of lignin; (vi) 1655 cm^-1^ peak associated with coniferalcohols of lignin. **(D)** Quantification of 1355 cm^-1^ to 1600 cm^-1^ signal intensity ratio for EW and LW spectra analysed. Throughout the figure light brown indicates EW and dark brown denotes LW. Datapoints for each analysed EW and LW zone or image are shown as individual dots on boxplots in **(B, D)**. Median is marked in the middle of boxplots in **(B, D)** with rectangle extending to the lower and upper quartile and whiskers showing the minimal and maximal values (excluding outliers not included in median quantitation). Results of one sample **(B)** and two sample **(D)** Student’s t-test are reported with * marking p ≤ 0.05 and *** marking p ≤ 0.001.

For microscopic analysis, both EW and LW cells were imaged (**Figure 4A**; **Figure S4**) within one section to allow for relative quantification of the lignin autofluorescence through analysis of average pixel intensity that was determined using a dedicated image processing script (**Script S1**). Ratio of mean intensity of signal was used to compare strength of lignin autofluorescence between EW and LW (**Figure 4B**). Our analysis indicated that, on average, the intensity of lignin autofluorescence is marginally, but significantly, greater in LW than in EW. This indicates that either (i) pine LW is more lignified than EW or (ii) LW and EW lignin has a different chemistry and a concomitant change in autofluorescence.

To provide a chemical insight on EW and LW lignin (and other secondary cell wall components), we obtained Raman spectra for pine EW and LW regions (**Figure 4C**). The spectra were dominated by signals from lignin (maximum peak intensity at 1600 cm^-1^, (i) on **Figure 4C**) and cellulose (two peak maxima at 1095 cm^-1^ and 1122 cm^-1^, (ii) and (iii) on **Figure 4C** respectively). Due to the presence of a large cell lumen, the density of the cell wall material within the EW region is considerably smaller than in LW. Therefore, it was necessary to standardise the spectra to be able to compare signal intensity for different cell wall features. Since our previous measurements indicated that the average cellulose content of EW and LW is not significantly different, we decided to use the cellulose 1122 cm^-1^ orientation independent signal (Gorzsás, 2017) (signal (iii) on **Figure 4C**) for the standardisation process. To this end, the intensity of the 1122 cm^-1^ EW signal was set to match that recorded for LW (**Figure 4C**). As a result, the intensity of signals in the standardised EW spectrum matched that observed for LW for most wavenumbers. For example, the orientation independent lignin signal at 1600 cm^-1^ in the standardised EW spectrum matched that recorded for LW. This observation is supported by our previous measurements (**Figure 4B**), which indicated that the lignin content in EW and LW is likely to be comparable. After standardisation, signal intensity was markedly different for one wavenumber region: 1300 – 1360 cm^-1^. The peak at 1335 cm^-1^ (marked as (iv) on **Figure 4C**) was stronger for EW than for LW. Previous work (Agarwal et al., 2011), assigned the 1335 cm^-1^ signal to aliphatic hydroxyls of lignin. Thus, to investigate if EW lignin may have higher content of aliphatic hydroxyls than the LW one, we decided to quantify the relative intensity of 1335 cm^-1^ signal to that of lignin 1600 cm^-1^ (**Figure 4D**). Our analysis indicated that the relative intensity of 1335 cm^-1^ signal is greater for EW than for LW. To identify further structural differences in lignin, we analysed other signals associated with the presence of coniferaldehydes (peak around 1620 cm^-1^ (v) on **Figure 4C**) and coniferalcohols (1655 cm^-1^, (vi) on **Figure 4C**) in pine EW and LW lignin (**Figure S5A**) (Agarwal and Ralph, 2008; Hänninen et al., 2011; Bock and Gierlinger, 2019). To this end, we again expressed the intensity of the 1655 cm^-1^ (**Figure S5B**) and 1620 cm^-1^ (**Figure S5C**) signals as a proportion of the 1600 cm^-1^ peak. This analysis indicated that LW appears to have a stronger coniferaldehyde signal than the EW and conversely, the EW has a greater coniferalcohol signal than the LW.

To provide further insight into this structural diversity between EW and LW lignin, we also obtained Raman spectra for the two wood types at a level of individual cell walls. In this analysis, we isolated spectra at a spatial resolution of 300 nm, positioned along a line spanning across the cell walls of two neighbouring tracheids. In subsequent analysis, we focused on the coniferaldehyde signal that showed minimal discrepancy between the EW and LW in the low magnification Raman analysis above (**Figures S5A, B**). Sub-micron examination of LW tracheids demonstrated that the coniferaldehyde signal is present only in some areas of the cell wall, suggesting that this type of lignin shows spatial heterogeneity within the cell wall (**Figures S6A, B**). Surprisingly, inspection of further areas indicated that the strong coniferaldehyde signal is only observed for spectra obtained from the LW cell walls, and that EW cell walls never produce a peak at this wavenumber in our analysis (**Figure S7**). Together with previous data, this Raman analysis indicates that the exact structure of lignin is different across the two different wood types. In particular, EW lignin may have a greater amount of aliphatic hydroxyls while the LW lignin seems to be composed of coniferaldehyde subunits to a greater extent than the EW one.

## 4 Discussion

In this manuscript we have looked in detail at the composition of earlywood (EW) and latewood (LW) secondary cell walls in *P. sylvestris*. Saccharification with an enzymatic cocktail (**Figures 1C, D**) demonstrated that both glucose and xylose release is greater from LW than it is from EW. Importantly, we also observed that enzymatic digestion of GGM releases a greater quantity of oligosaccharides from LW than from EW (**Figures 2** and **S2A**) and that quantity of mannose released by mannanase from LW digestion is also greater than that from EW (**Figure S2B**). Given the high degree of similarity in composition between the two wood types (**Figures 1A, B** and **Figure 3**), we propose therefore that in *P. sylvestris* LW is more accessible to enzymatic digestion than the EW.

To understand the origin of the observed differences in recalcitrance, we analysed the structure of hemicelluloses in EW and LW of pine (**Figures 2**, **Figure 3**). Hemicelluloses are proposed to interact with cellulose and lignin (Simmons et al., 2016; Busse-Wicher et al., 2016b; Grantham et al., 2017; Nishimura et al., 2018; Terrett and Dupree, 2019; Terrett et al., 2019; Crowe et al., 2021) and as such they can contribute to the maintenance of biomass properties and recalcitrance. Our experiments indicate that LW xylan appears to be more heavily branched with arabinose and GlcA compared to EW (**Figures 3B, C**). Mannose is a dominant non-cellulosic monosaccharide in wood of *P. sylvestris* (**Figure 1**) and other pine species like *Pinus radiata* (Reyes et al., 2013), therefore it is important to also study contribution of GGM to recalcitrance. Digestion of EW and LW biomass with two different mannanases suggests that the structure of GGM in EW and LW may be very similar (**Figures 2A, B**). Together with the outcomes of saccharification experiments, these results indicate that despite having some structural features associated with more recalcitrant biomass type, for example higher degree of xylan glucuronidation, the LW biomass is less resistant to enzymatic digestion than the EW one. Therefore, it is possible that the influence of hemicellulose structure on recalcitrance of EW and LW biomass may be masked by other features of cell wall molecular architecture, such as the lignin content or structure.

To evaluate the structural features of EW and LW lignin, we obtained Raman spectra for pine cores and separated the data for the two wood types (**Figure 4C**). Following standardisation of the spectra, necessary due to different density of the cell wall material, we observed that EW has markedly increased signal associated with aliphatic hydroxyls of lignin (Agarwal et al., 2011) when compared to LW (**Figure 4D**). Aliphatic hydroxyl groups on lignin are prevalent in lignin bonded *via* linkages such as β-O-4, α-O-4 or β-5 (Ralph et al., 2019). On the other hand, lignin linked through bonds such as β-β has a smaller content of hydroxyl groups in the polymer. Interestingly, previous work has linked increased presence of β-β lignin linkages with higher saccharification yield (Shi et al., 2016). This could be due to more compact and hydrophobic nature of the β-β lignin which may have a lower capacity to interact with cell wall polysaccharides (Li et al., 2016). Therefore, the increased intensity of aliphatic hydroxyl signals in EW, when compared to LW, is likely a hallmark of lignin linkages which are associated with higher biomass recalcitrance.

We additionally found that LW has a stronger coniferaldehyde Raman signal than EW (**Figures S5A, B**, **Figure S7**). Conversely, the signal for coniferalcohols was greater in EW than in LW (**Figure S5C**). Consistent with our data, previous work has associated higher coniferaldehyde content with lower biomass recalcitrance to enzymatic digestion (Yamamoto et al., 2020). The reasons for this are not clear but may involve alterations to lignin-polysaccharide interactions due to presence of these coniferaldehydes. Cinnamaldehydes, which give rise to coniferaldehyde lignin in softwood, do not undergo nucleophile attack during resolution of the quinone methide intermediate as part of lignin polymerisation. This means, in lignin with higher coniferaldehyde content, hemicelluloses cannot act as nucleophile donors and it is possible they may not be incorporated into the lignin polymer as part of lignin carbohydrate complexes (LCCs) (Terrett and Dupree, 2019). Therefore, we postulate that LW hemicelluloses do not form LCCs as readily as the EW ones. LW xylan, despite being more heavily branched with GlcA that can participate in LCC formation (Lyczakowski et al., 2017), might not be forming covalent bonds with lignin to the same extent as the less glucuronidated EW xylan. Similarly, the LW GGM might not be able to form ether linkages to lignin (Nishimura et al., 2018) as readily as the EW one.

**Figure 5:**
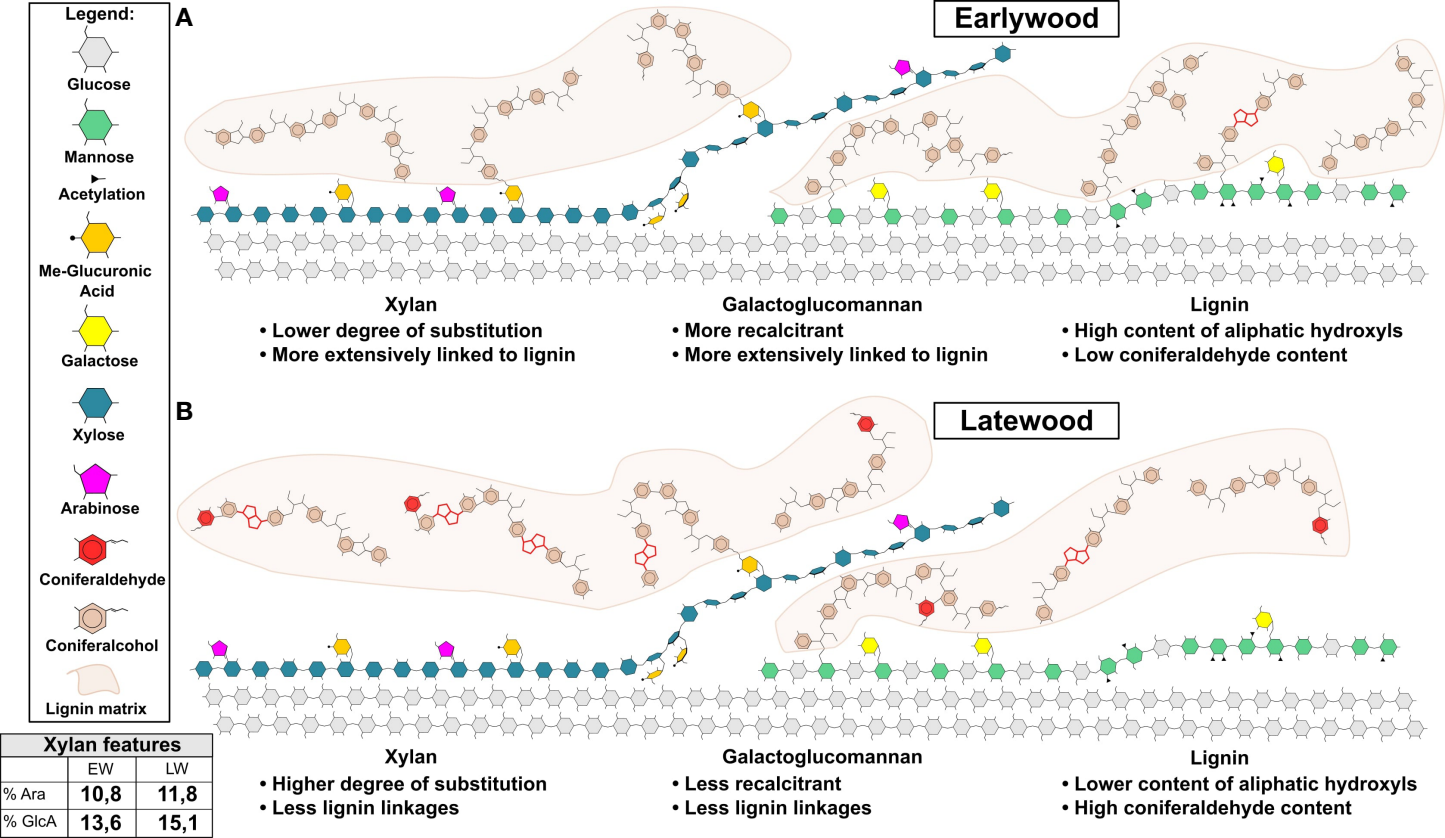
Model of earlywood (EW) and latewood (LW) secondary cell wall molecular architecture. Cellulose, xylan, GGM and lignin are shown. Model highlights the main differences in the features of softwood’s EW **(A)** and LW **(B)** secondary cell wall. These include: (1) greater extent of lignin cross-linking to hemicelluloses in EW then in LW; (2) presence of lignin linkages favouring maintenance of aliphatic hydroxyls in EW and reduction in such linkages in LW and (3) presence of coniferaldehydes in LW lignin only. In addition to that, xylan and mannan regions compatible and incompatible with cellulose binding are shown for both wood types in line with current knowledge on softwood cell wall molecular architecture described in the literature (Busse-Wicher et al., 2016a; Martínez-Abad et al., 2017; Yu et al., 2018; Terrett et al., 2019; Martínez-Abad et al., 2020; Lyczakowski et al., 2021). Lignin matrix (in light brown) is shown and only parts of lignin structure are drawn in detail. These highlight coniferaldehydes and β-β linkages in red. For ease of depiction xylan marked on figure lacks reducing end oligosaccharide which is likely to exist in pine since it was detected in another conifer: spruce (Andersson et al., 1982). A table is provided with quantitative data on xylan branching with arabinose and MeGlcA as measured through PACE analysis in our work.

Our experiments enable us to provide a summary of structural features distinguishing the EW and LW cell walls (**Figure 5** and **Table 1**) in *Pinus sylvestris*. Biochemical characterisation of wood cell walls (**Figures 1A, B**) showed that hemicellulose composition and cellulose content are largely similar in EW and LW, with only xylan content marginally higher in EW. Analysis of xylan structure with PACE (**Figure 3**) showed that the polysaccharide is more branched in LW than in EW. For GGM, the structure of the polysaccharide visualized with PACE is the same in both wood types (**Figures 2A, B**). Importantly, our saccharification experiments (**Figures 1C, D**, **Figures 2C, D** and **Figure S2**) showed that LW cellulose, xylan and GGM are more accessible to enzymatic degradation than EW ones. Absence of major differences in cell wall polysaccharides led us to propose a hypothesis that structural features of EW and LW lignin may underline the observed differences in accessibility of the two wood types to enzymatic digestion. To evaluate this, we performed Raman analysis which showed that EW lignin has more free aliphatic hydroxyls (**Figures 4C, D**) than LW one and that LW lignin has a substantially higher coniferaldehyde content than the EW one (**Figures S5** and **S6**). Together, this suggests that EW lignin has a greater capacity for LCC formation with wall polysaccharides than the LW polymer. All structural features discussed above are presented on **Figure 5** and we believe that this model may guide future research into tree breeding and wood utilisation, principally aimed at improving the efficiency of wood residue processing and conversion.

**Table 1:** Summary of molecular features of the different components of EW and LW cell walls.

Cell wall component	EW features	LW features
*Cellulose*	EW and LW cell wall cellulose content is similar but shows significant variation between different *Pinus sylvestris* individuals.	
*Xylan*	EW may have a higher xylan content than the LW. The EW xylan shows a lower degree of substitution than the LW one.	LW xylan has a higher degree of arabinosylation and glucuronidation than the EW one, what may stabilise interaction of this hemicellulose with the cellulose microfibril (Pereira et al., 2017) in LW cell walls.
*GGM*	GGM content and structure (i.e. the mannose to glucose ratio, the degree of galactosylation) like that in LW, but the polysaccharide is more recalcitrant to enzymatic degradation.	GGM content and structure (i.e. the mannose to glucose ratio, the degree of galactosylation) is like in EW GGM, but the polysaccharide is significantly less recalcitrant to enzymatic hydrolysis by mannanases than the EW one. This may be associated with a lower extent of lignin cross-linking to GGM through ether linkages in LW than in the EW.
*Lignin*	EW lignin is characterised by a high content of aliphatic hydroxyls. This may be originating from a high proportion of β-O-4, α-O-4 or β-5 linkages. EW lignin has a low coniferaldehyde content.	LW lignin has a lower content of aliphatic hydroxyls than the EW one, what may be a result of higher content of β-β lignin linkages in this wood type. LW lignin has a high coniferaldehyde content and the coniferaldehydes are distributed throughout the LW cell wall. This may impair formation of linkages with hemicelluloses (Terrett and Dupree, 2019) and reduced recalcitrance of LW to enzymatic saccharification.

Similar saccharification data from biomass of Douglas fir, pre-treated with dilute acid, indicated that latewood is less recalcitrant to enzymatic hydrolysis than earlywood (Zhang et al., 2014). This difference was lost when the biomass was pretreated with sulfite, which is more suited for lignin removal. This supports our observations that lignin may underline the difference in susceptibility to enzymatic digestion observed for EW and LW. Composition and hemicellulose structure has also been examined for EW and LW cell walls of Japanese cedar (Kurata et al., 2018). Again, supporting our data, the cedar analysis did not reveal major differences in monosaccharide composition of EW and LW hemicelluloses. Interestingly, the degree of LW xylan branching with GlcA and Ara in Japanese cedar is smaller than that of EW. This suggests that cell wall structure may show some inter-specific variation within the gymnosperm clade.

For lignin, the observed structural differences may originate from variable conditions of lignification or differences in the activity of lignin biosynthesis machinery between EW and LW (Tobimatsu and Schuetz, 2019). Indeed, previous work indicated variation in the rate of lignification (Antonova et al., 2014) and monolignol composition (Antonova et al., 2019) between pine EW and LW, what could contribute to the observed variation in lignin structure, this could be due to factors such as pH of lignification, monolignol type or supply rate may influence the structure of the resultant lignin polymer (Demont-Caulet et al., 2010; Tobimatsu et al., 2010; Hwang et al., 2015; Warinowski et al., 2016; Shigeto et al., 2018). Other putative factors include different capacities of peroxidases and laccases to oxidise the monolignools or polymeric substrates of lignification (Demont-Caulet et al., 2010; Hwang et al., 2015; Warinowski et al., 2016). Since a growing body of evidence suggests that laccases and peroxidases have different localisation in the plant cell wall (Chou et al., 2018), it is not inconceivable that the enzymes would also exhibit temporal differences in activity which can contribute to observed changes in lignin structure.

Finally, it is important to evaluate the potential impact of our analysis on the industrial utilisation of pine biomass. Central to this is our observations that pine EW is more recalcitrant to enzymatic digestion than LW. This is particularly clear for GGM, where the release of oligosaccharides is, on average, more than 2 times more efficient from LW than from EW. This difference in recalcitrance is an important consideration for utilisation of pine biomass with high EW content such as juvenile wood, which is increasingly being harvested in softwood plantations (Zobel, 2004). To improve the processing of such earlywood-rich feedstocks, it may be necessary to modify pretreatment protocols to increase lignin degradation or alternatively attempt to develop mannanase enzymes capable of digesting EW GGM efficiently. Addition of enzymes targeting cross-linking between hemicelluloses and lignin, such as glucuronyl esterases from carbohydrate esterase family 15 (d’Errico et al., 2016), may also have a beneficial effect on the processing of earlywood-rich pine feedstocks. In the long term, analysis of biological origins of the observed variation in the structure of softwood lignin may provide breeding or genetic engineering targets for improving the efficiency of processing pine feedstocks.

In summary, we present here the first comprehensive overview of cell wall molecular architecture in pine EW and LW. In our analysis we evaluated the monosaccharide composition, cellulose content, xylan, mannan and lignin structure for both wood types in *P. sylvestris.* We observed that pine EW polysaccharides have higher recalcitrance to enzymatic digestion than the LW ones. This difference may originate from the variation in lignin structure which influences the accessibility of cell wall polysaccharides to enzymatic hydrolysis. Our data enabled us to propose a model of the molecular architecture of earlywood and latewood secondary cell walls. As such, our work will facilitate development of improved processing strategies for softwood or can guide conifer breeding programmes aimed at generation of more easily digestible woody biomass.

## Data availability statement

The original contributions presented in the study are included in the article/**Supplementary Material**. Further inquiries can be directed to the corresponding author.

## Author contributions

AL: Formal analysis, Investigation, Methodology, Resources, Visualization, Writing – review & editing, Data curation, Validation. RW: Data curation, Formal analysis, Investigation, Methodology, Project administration, Resources, Visualization, Writing – original draft, Validation. DL: Formal analysis, Investigation, Methodology, Supervision, Validation, Writing – review & editing. MB: Investigation, Methodology, Software, Writing – review & editing. KK: Formal analysis, Methodology, Resources, Validation, Writing – review & editing. MP: Formal analysis, Methodology, Resources, Writing – review & editing. JL: Conceptualization, Formal analysis, Funding acquisition, Investigation, Methodology, Project administration, Supervision, Writing – original draft, Data curation, Resources, Validation.
