# Supplementary material for: Structural differences of cell walls in earlywood and latewood of Pinus sylvestris and their contribution to biomass recalcitrance
Source: Front Plant Sci. 2023 Dec 8;14:1283093. doi: 10.3389/fpls.2023.1283093 (PMC10749964; doi:10.3389/fpls.2023.1283093)
Supplement: Supplementary file 1 [file DataSheet_1.pdf]

Supplementary Material

Supplementary Material for

Structural differences of cell walls in earlywood and latewood of *Pinus sylvestris* and their contribution to biomass recalcitrance

authored by

Aleksandra Liszka, Raymond Wightman, Dariusz Latowski, Matthieu Bourdon, Kristian B. R. M. Krogh, Marcin Pietrzykowski, Jan J. Lyczakowski*

*corresponding author: Jan J Lyczakowski (jan.lyczakowski@uj.edu.pl)

This file includes the following figures and a script:

Fig. S1: Scheme describing the sample preparation process and monosaccharide separation on HPLC.
Fig. S2: Oligosaccharides released from EW and LW after digestion of biomass not treated with NaOH with mannanase GH5 and quantitative measurement of mannose release after GH5 digest.
Fig. S3: Annotation of oligosaccharides released with xylanase GH11 from EW and LW cell walls.
Fig. S4: Further images of lignin autofluorescence in EW and LW.
Fig. S5: Coniferaldehyde and coniferalcohol Raman signals in pine EW and LW.
Fig. S6: Spatial heterogeneity in the coniferaldehyde signal in latewood cell walls.
Fig. S7: Absence of coniferaldehyde signals in earlywood cell walls.
Script S1: Macro used for the comparative quantification of lignin autofluorescence.

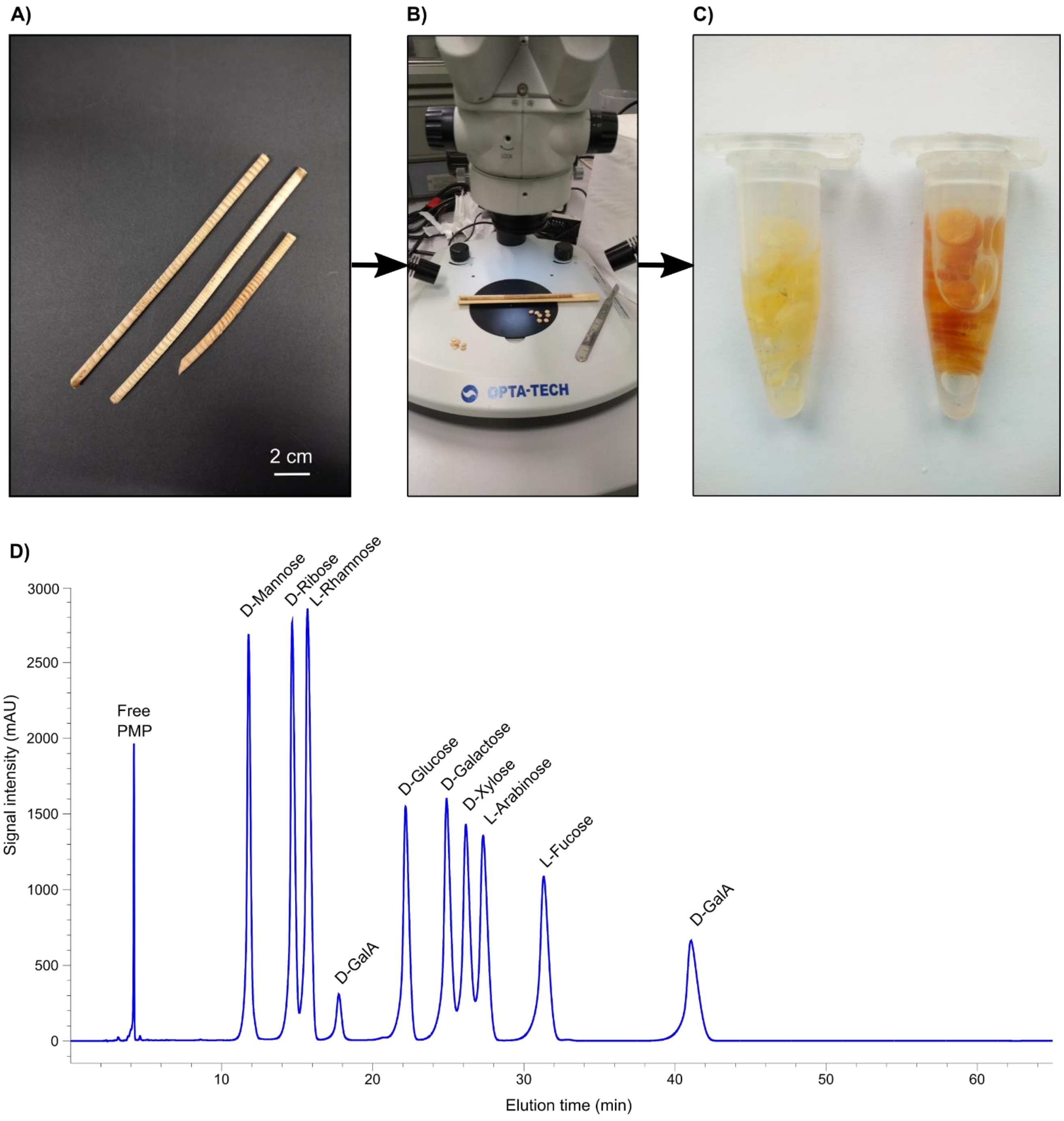

Fig. S1. Scheme describing the sample preparation process and monosaccharide separation on HPLC. A) Cores of *Pinus sylvestris* isolated with an increment borer from trees. B) Sectioning of *Pinus sylvestris* cores under a stereomicroscope. C) Individual samples of EW and LW prior to milling and AIR preparation. D) Separation of standard monosaccharides on HPLC, D-galacturonic acid abbreviated to D-GalA.

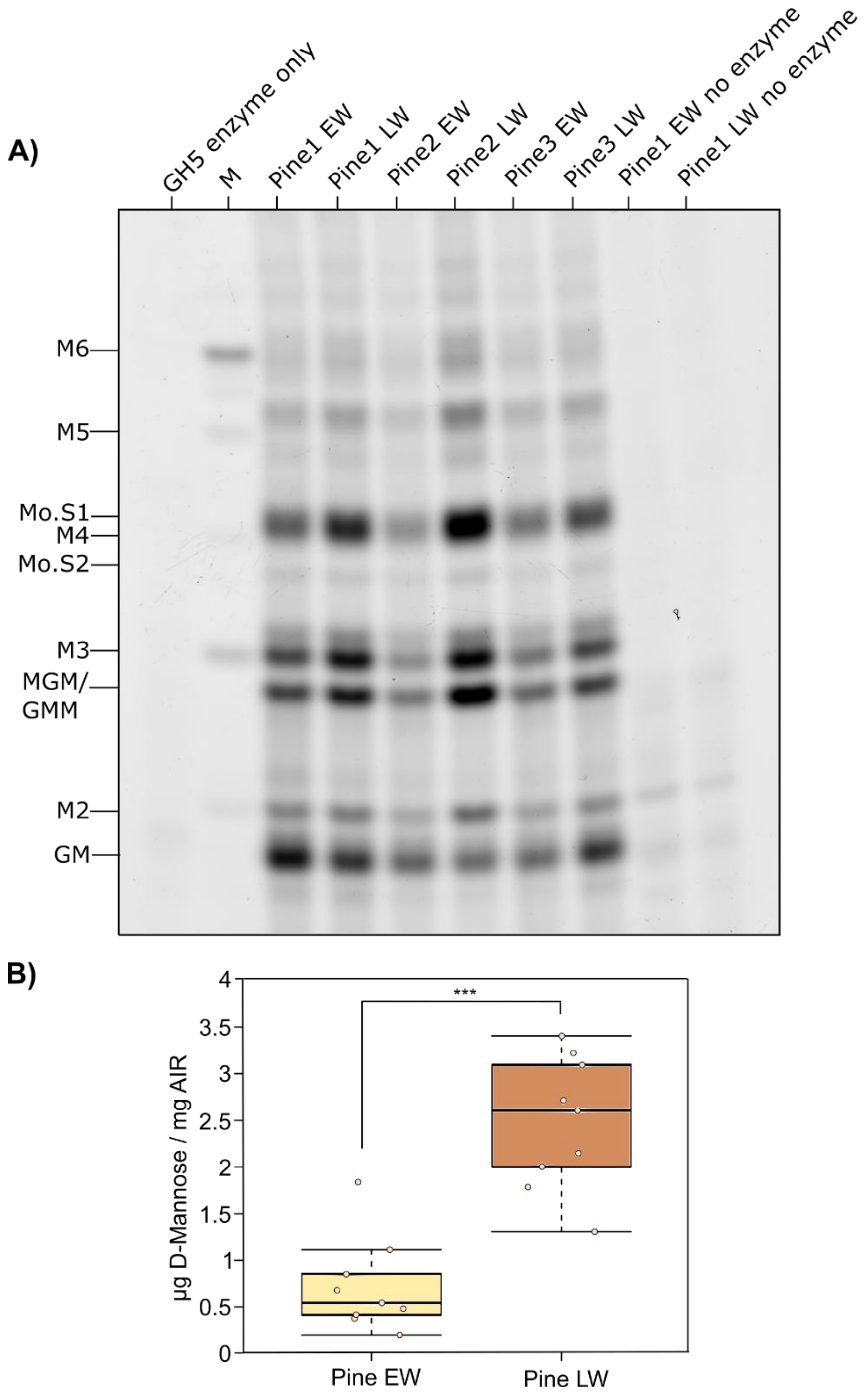

Fig. S2. Oligosaccharides released from EW and LW after digestion of biomass not treated with NaOH with mannanase GH5 and quantitative measurement of mannose release after GH5 digest. A) PACE of EW and LW AIR digestion with GH5 mannanase. AIR used for this digestion was not treated with NaOH prior to the addition of the enzyme. Fuzziness of bands is likely associated with variation in the acetylation pattern of the oligosaccharides. M denotes the M1 to M6 mannan migration standard. Biomass not digested with mannanase and mannanase only containing samples were analysed as controls. B) Total quantity of mannose released as either monosaccharide or oligosaccharide by GH5 from EW and LW not treated with NaOH. Analysis performed for three individual pine trees in triplicate. Results of Student's t-test are denoted with *** indicating $p<0.0001$.

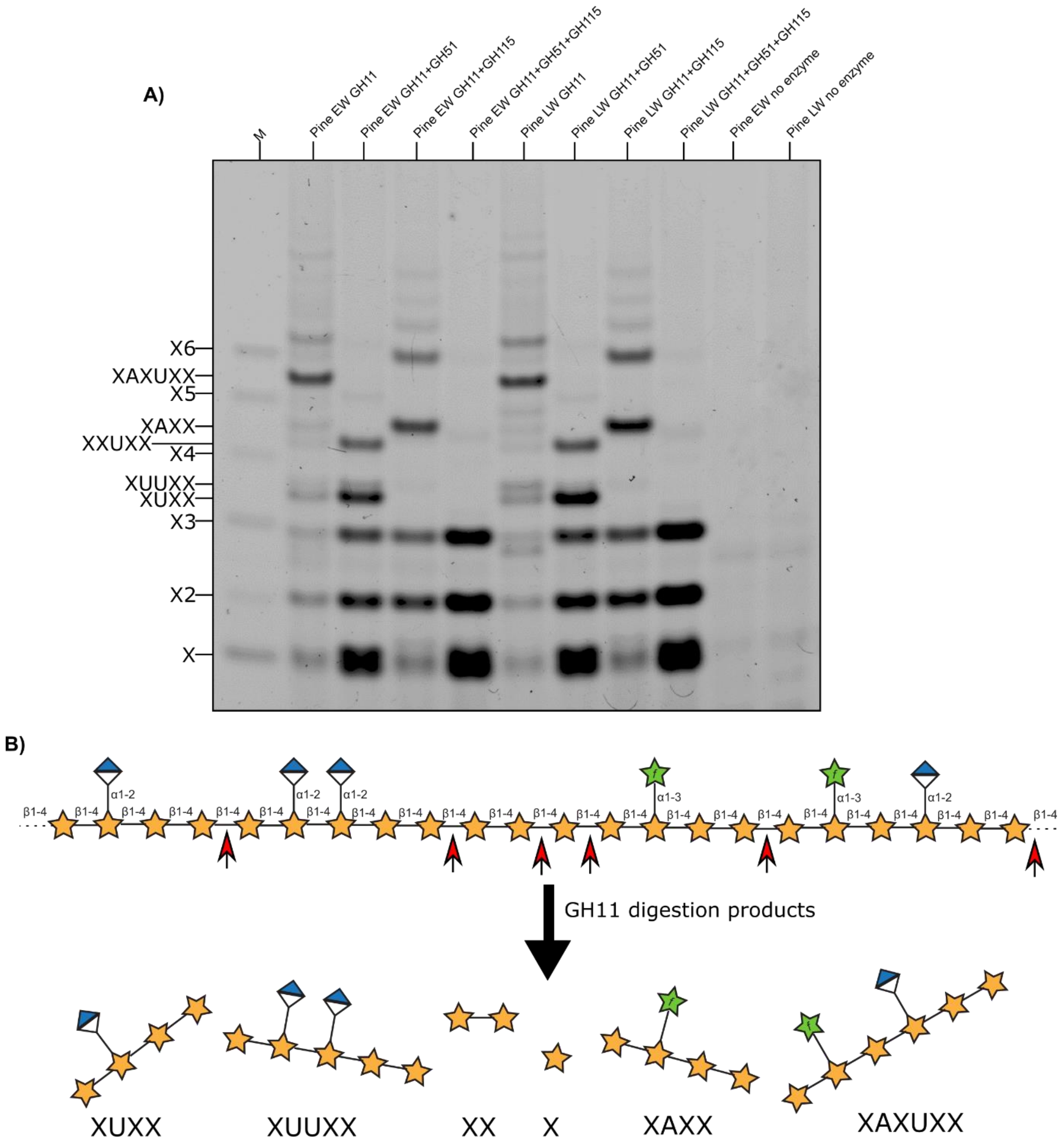

Fig. S3. Annotation of oligosaccharides released with xylanase GH11 from EW and LW cell walls. A) PACE of EW and LW AIR digestion with GH11 xylanase alone, GH11 and GH51 arabinofuranosidase, GH11 and GH115 alpha-glucuronidase and GH11 together with both GH51 and GH115. M denotes a lane with xylan migration standard. Glucuronidated and/or arabinosylated oligosaccharides are labelled. Digestion of the EW and LW AIR with both GH51 and GH11 resulted in changes in the PACE band migration pattern compared to GH11 alone. The two unknown structures, S1 and S2, disappeared and the intensity of other bands, in particular that of XUXX, increased. S1 and S2, therefore, likely contain arabinose substitutions. The XUXX and XUUXX structures disappeared after treatment with both GH115 glucuronidase and GH11. This is consistent with both structures being branched with only GlcA which are removed by GH115. Interestingly, the oligosaccharide S1 also disappeared while the intensity of oligosaccharide S2 increased and points to oligosaccharide S1

containing arabinose and GlcA branches while oligosaccharide S2 is decorated with just arabinose. Therefore, in agreement with previously published information for spruce (*27*), we annotated pine structure S1 as being XAXUXX and oligosaccharide S2 as XAXX. To further validate our annotation, we digested EW and LW AIR with GH11, GH51 and GH115. This led to a complete removal of oligosaccharides S1, S2, XUUXX and XUXX. These were replaced with xylose, xylobiose and xylotriose, confirming that all the GH11 digestion products released from EW and LW AIR are branched with arabinose and/or GlcA decorations. Moreover, we observed no differences in the presence/absence of bands between EW and LW. B) Scheme of GH11 digestion of conifer xylan. Digestion points are denoted with red arrows. Resulting oligosaccharides are annotated, with X indicating an unsubstituted xylose (orange star), A indicating a xylose substituted with 3 linked arabinofuranose (green star) and U indicating a xylose with 2 linked MeGlcA (blue-white diamond).

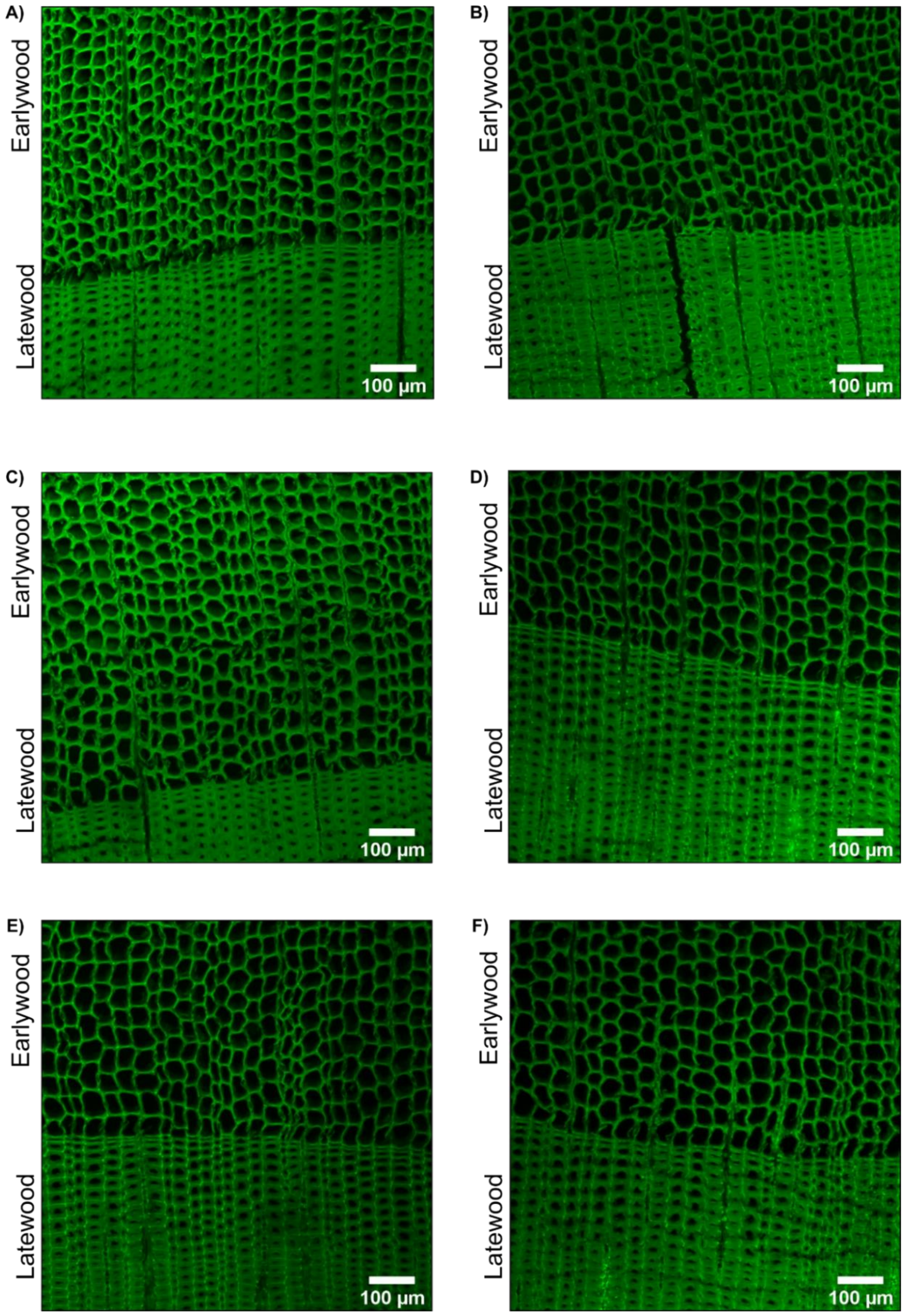

Fig. S4. Further images of lignin autofluorescence in EW and LW. A) to F) shows further confocal images of lignin autofluorescence in pine wood sections.

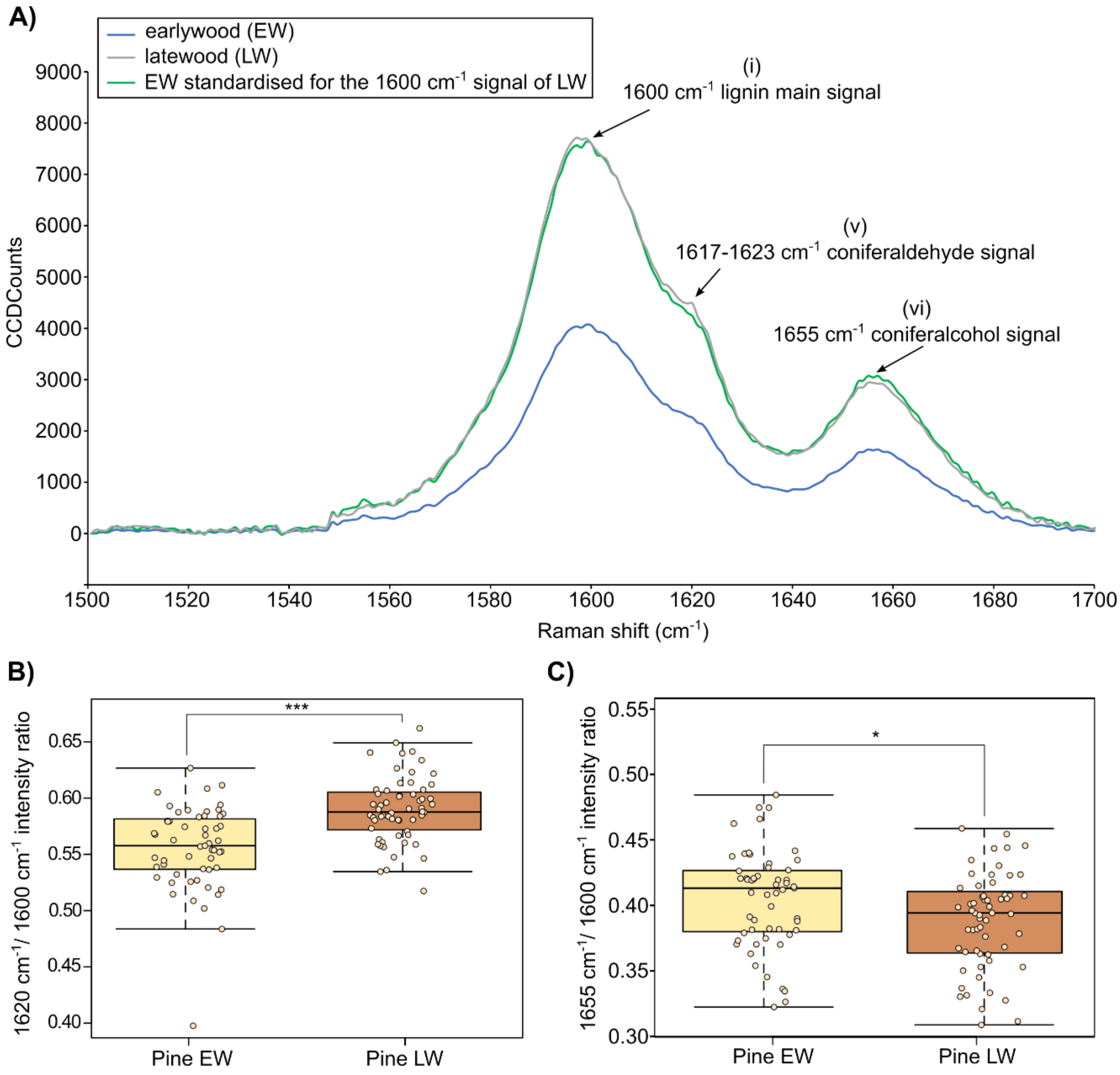

Fig. S5. Coniferaldehyde and coniferalcohol Raman signals in pine EW and LW. A) Zoom in of the averaged Raman spectrum in the 1500 cm^{-1} to 1700 cm^{-1} range, showing the earlywood (EW, blue), latewood (LW, grey) and EW standardised for the 1600 cm-1 signal of LW (green) spectra. Panel highlights the main lignin peak (i), the coniferaldehyde signal (v) and the coniferalcohol (vi) one. B) Ratio of the 1620 cm-1 to 1600 cm-1 signals in pine EW and LW. This shows the intensity of the coniferaldehyde signal as a proportion of the main lignin peak. C) Ratio of the 1655 cm-1 signal to the 1600 cm-1 on for pine EW and LW. This shows the intensity of the coniferalcohol signal as a proportion of the main lignin peak. In both B) and C) *** indicate $p<0.0001$ and * denotes $p<0.05$.

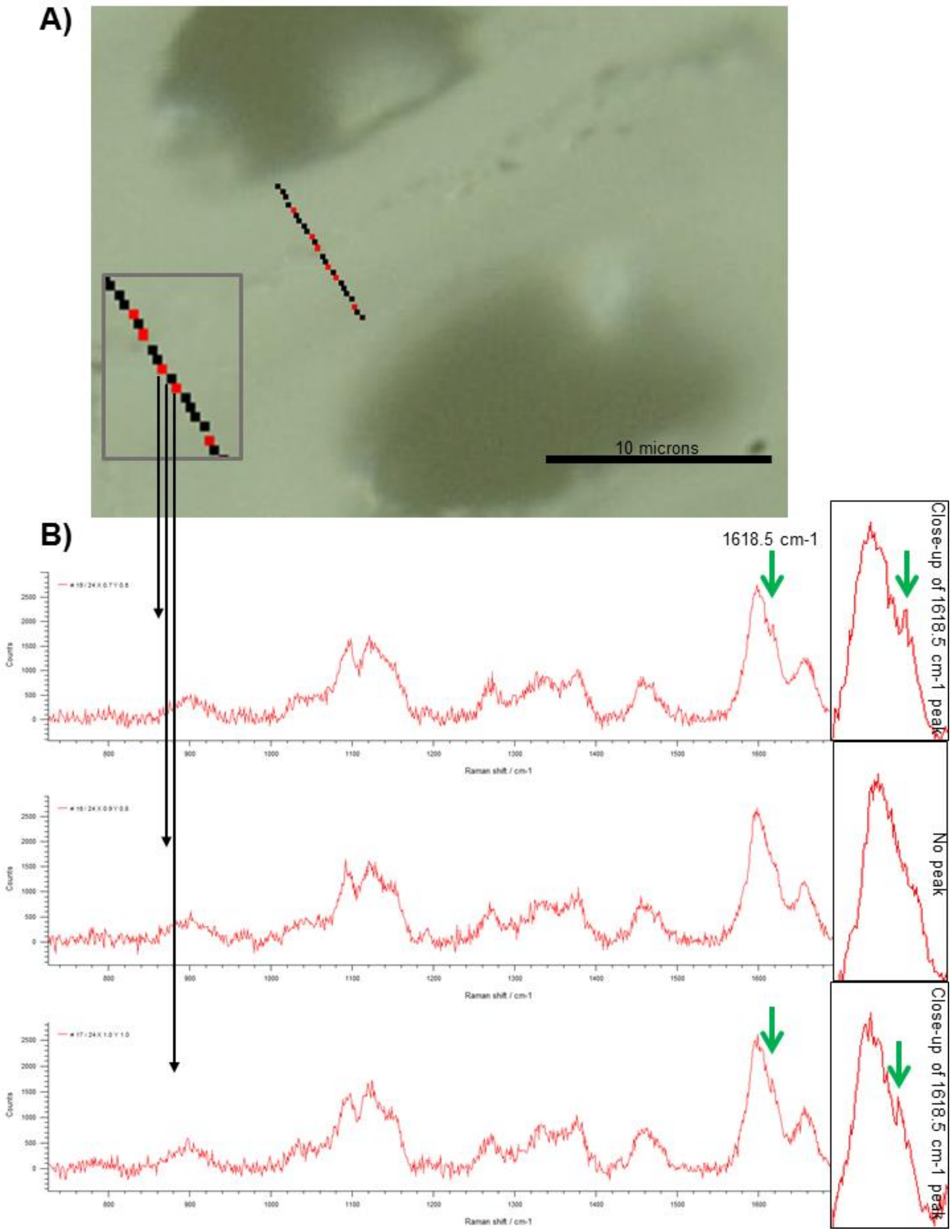

Fig. S6. Spatial heterogeneity in the coniferaldehyde signal in latewood cell walls. Analysis of the presence/absence of the Raman 1618.5 cm^{-1} peak in latewood. A) The image shows two latewood cells. A linescan of point spectra at 300 micron intervals was taken that covers cell walls from two neighbouring cells. Sites along the line where a peak at 1618.5 cm^{-1} is observed are shown as red pixels

(generated automatically, see methods). A magnified view (inset) of part of a cell wall shows two red pixels (calculated as having a peak) flanking a black (no calculated peak) pixel. The corresponding Raman spectra are shown in B). The sites overlayed by the red pixels clearly exhibit the distinct peak at 1618.5 cm^{-1} with the site between (middle spectrum) exhibiting no peak. This sub-micron heterogeneity of the 1618.5 cm^{-1} peak (red) interspersed with sites without the peak (black) were observed in all latewood experiments (n=3, SI Figure 7 left). The same analysis of earlywood linescans (n=3, SI Figure 7 right) found no red pixels (meaning no 1618.5 cm^{-1} peaks and therefore no spatial heterogeneity).

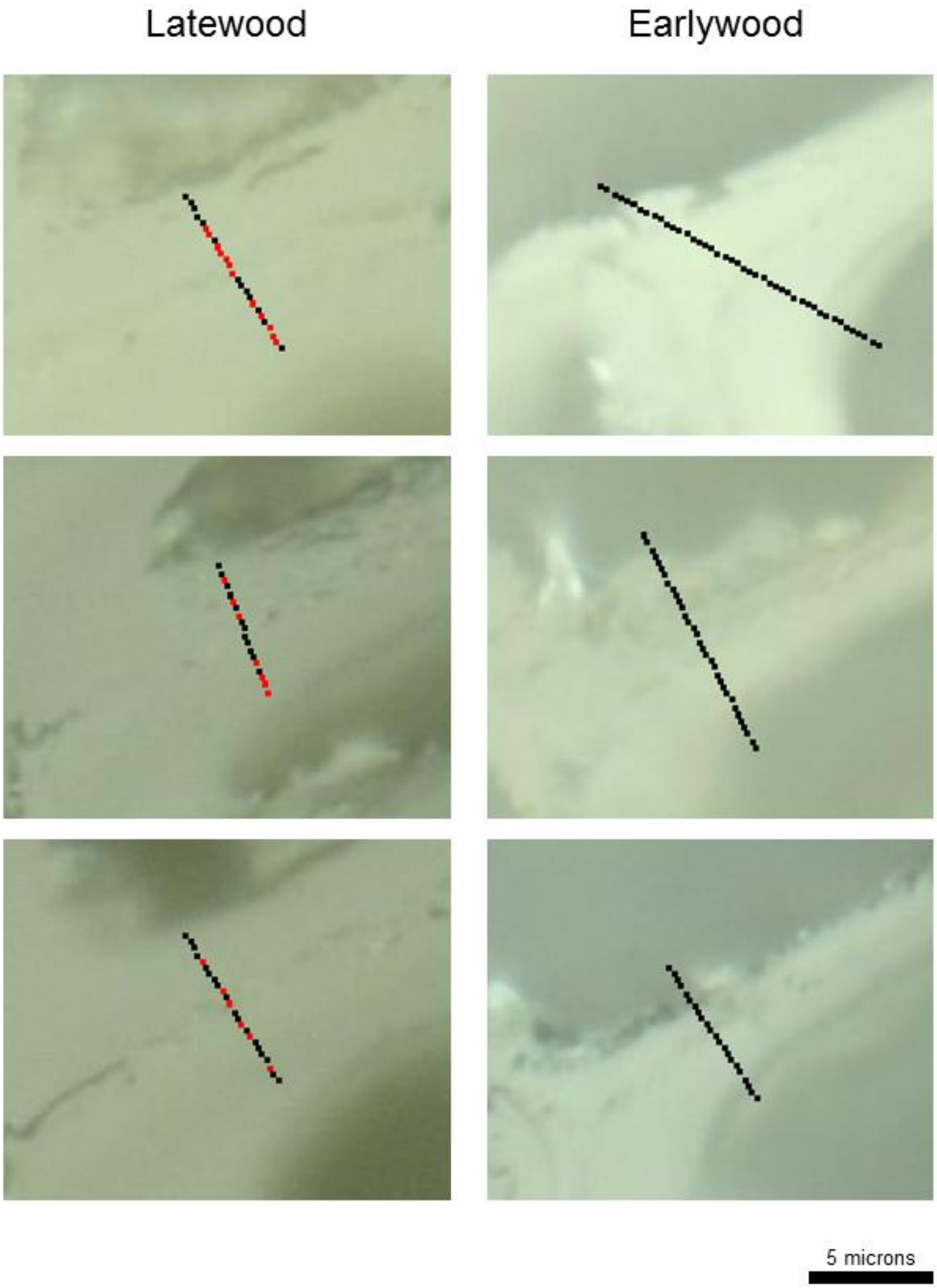

Fig. S7. Absence of coniferaldehyde signals in earlywood cell walls. Spatial heterogeneity in terms of presence of 1618.5 cm^{-1} peak is only observed in latewood (red pixels on left hand side). Shown are three independent Raman line maps each of late and earlywood (right hand side) cells. Scale bar is provided and applies to all images taken.

Script S1. Macro used for the comparative quantification of lignin autofluorescence.

```
"Stack to MAX, scale bar, save composite and individual channels "{
dir=getDirectory("Choose directory");
name=File.nameWithoutExtension;
print(dir);
AnalysisDir= dir+"/Analysis and splitted files/";
print(AnalysisDir);
File.makeDirectory(AnalysisDir);
path = AnalysisDir+name;
run("Z Project...", "projection=[Max Intensity]");
saveAs("Tiff", path+"_MAX_Tiff");
makeRectangle(1359, 120, 600, 24);
setTool("rectangle");
waitForUser("Create a rectangle selection to adjust Scale bar location");
run("Scale Bar...");
run("Make Composite");
saveAs("Jpeg", path+"_MAX_Composite");
imageTitle=getTitle();
run("Split Channels");
selectWindow("C1-"+imageTitle);
saveAs("Jpeg", path+"_MAX_lignin");
selectWindow("C2-"+imageTitle);
saveAs("Jpeg", path+"_MAX_Brightfield");
run("Close");
selectWindow("C1-"+imageTitle);
run("Duplicate...", " ");
run("Enhance Local Contrast (CLAHE)", "blocksize=127 histogram=256 maximum=3
mask=*None*");
setAutoThreshold("Li dark")
run("Create Selection");
roiManager("Add");
saveAs("Selection", path+"_ROI_all_wood");
roiManager("Select", 0);
run("Histogram", "bins=256 x_min=0 x_max=65536");
saveAs("Jpeg", path+"_all_wood_intensity_histogram");
selectWindow("C1-"+imageTitle);
run("Duplicate...", " ");
setTool("freehand");
waitForUser("Draw manually EARLY WOOD ROI:\nDrawing inside current selection while
pressing Alt creates a hole removing content from the ROI,\nDrawing outside current selection while
pressing Shift creates new content")
run("Clear Outside", "stack");
run("Enhance Local Contrast (CLAHE)", "blocksize=127 histogram=256 maximum=3
mask=*None*");
setAutoThreshold("Li dark")
run("Create Selection");
roiManager("Add");
```

```
saveAs("Selection", path+"_ROI_early_wood");
selectWindow("C1-"+imageTitle);
run("Duplicate...", " ");
setTool("freehand");
waitForUser("Draw manually LATE WOOD ROI: Drawing inside current selection while pressing
Alt creates a hole removing content from the ROI, Drawing outside current selection while pressing
Shift creates new content")
run("Clear Outside", "stack");
run("Enhance Local Contrast (CLAHE)", "blocksize=127 histogram=256 maximum=3
mask=*None*");
setAutoThreshold("Li dark")
run("Create Selection");
roiManager("Add");
saveAs("Selection", path+"_ROI_late_wood");
selectWindow("C1-"+imageTitle);
roiManager("Select", 1);
waitForUser("Check that ROI match well the area to be measured");
run("Set Measurements...", "area mean standard modal min display redirect=None decimal=2");
run("Measure");
roiManager("Select", 2);
waitForUser("Check that ROI match well the area to be measured");
run("Set Measurements...", "area mean standard modal min display redirect=None decimal=2");
run("Measure");
selectWindow("C1-"+imageTitle);
roiManager("Select", 1);
run("Histogram", "bins=256 x_min=0 x_max=65536");
saveAs("Jpeg", path+"_early_wood_intensity_histogram");
selectWindow("C1-"+imageTitle);
roiManager("Select", 2);
run("Histogram", "bins=256 x_min=0 x_max=65536");
saveAs("Jpeg", path+"_late_wood_intensity_histogram");
roiManager("Delete");
roiManager("Delete");
run("Close All");
}
```
